# Magnitude and Correlates of Intimate Partner Violence against Women and Its Outcome in Southwest Ethiopia

**DOI:** 10.1371/journal.pone.0036189

**Published:** 2012-04-25

**Authors:** Kebede Deribe, Biruk Kebede Beyene, Anbessu Tolla, Peter Memiah, Sibhatu Biadgilign, Alemayehu Amberbir

**Affiliations:** 1 Department of General Public health, Jimma University, Jimma, Ethiopia; 2 Fayyaa Integrated Development Organization, Addis Ababa, Ethiopia; 3 University of Maryland School of Medicine-Institute of Human Virology, Baltimore, Maryland, United States of America; 4 Departments of Epidemiology and Biostatistics, College of Public Health and Medical Science, Jimma University, Jimma, Ethiopia; 5 School of Public Health, Addis Ababa University, Addis Ababa, Ethiopia; 6 Division of Epidemiology & Public Health, the University of Nottingham, Nottingham, United Kingdom; Tulane University, United States of America

## Abstract

**Background:**

Intimate Partner Violence (IPV) is a major public health problem with serious consequences. This study was conducted to assess the magnitude of IPV in Southwest Ethiopia in predominantly rural community.

**Methods:**

This community based cross-sectional study was conducted in May, 2009 in Southwest Ethiopia using the World Health Organization core questionnaire to measure violence against women. Trained data collectors interviewed 851 ever-married women. Stata version 10.1 software and SPSS version 12.0.1 for windows were used for data analysis.

**Result:**

In this study the life time prevalence of sexual or physical partner violence, or both was 64.7% (95%CI: 61.4%–67.9%). The lifetime sexual violence [50.1% (95% CI: 46.7%–53.4%)] was considerably more prevalent than physical violence [41.1% (95%:37.8–44.5)]. A sizable proportion [41.5%(95%CI: 38.2%–44.8%)] of women reported physical or sexual violence, or both, in the past year. Men who were controlling were more likely to be violent against their partner.

**Conclusion:**

Physical and sexual violence is common among ever-married women in Southwest Ethiopia. Interventions targeting controlling men might help in reducing IPV. Further prospective longitudinal studies among ever-married women are important to identify predictors and to study the dynamics of violence over time.

## Introduction

Violence against women (VAW) is recognized as a significant public health problem, with grave consequences for women's physical, mental, sexual, and reproductive health [1], [2], [3]. It violates human rights and incurs substantial social, economic and health costs. Though violence occurs in different forms and settings including workplace, school and community, violence at home ‘domestic violence’ is considered as the most pervasive form [3], [4], [5]. The most common type of violence against women is intimate partner violence (IPV), which refers to any behavior within an intimate relationship that causes physical, psychological or sexual harm to those in the relationship [6]. There is an association between perpetration of rape and HIV sero-status. Recently published finding reported that in South African studies, 29.6% (358/1211) of men disclosed rape perpetration,5.2% (63/1208) had been raped in the past year and 30.7% (362/1180) of ever partnered men had been physically violent towards an intimate partner on more than one occasion [7]. A project evaluation involving two urban and three rural primary care facilities, over 4–8 weeks primary care providers screened adult women for a history of IPV within the previous 24 months, they found that emotional (139,82.7%), physical (115, 68.5%), sexual (72, 42.9%) and financial abuse (72, 42.9%) were common and 114 (67.9%) had a high or severe risk of harm [8].

According to a WHO multi-country study 15% to 71% of women had experienced physical or sexual partner violence or both in their lifetime. Furthermore, between 4% and 54% of respondents reported physical or sexual partner violence, or both, in the past year [9]. The same study revealed that in a province in Central Ethiopia 70.9% of women had experienced physical or sexual partner violence or both in their lifetime and 53.7% of respondents reported physical or sexual partner violence, or both, in the past year [9]. In other studies Northwest Ethiopia, South Central and Southwest Ethiopia of life time prevalence of about 52% physical or sexual violence was reported [10], [11], [12]. The discrepancies among the WHO's and other studies indicates the importance of site specific studies to document the prevalence and forms of violence in Ethiopia.

In the Ethiopian legal context, there is no consolidated law on violence against women. There are, however, provisions scattered in different sections of the 1957 Penal Code outlawing rape, abduction, early marriage, trafficking in women and other forms of crimes linked to sexuality. The 1957 Penal Code ignores other major forms of violence including domestic violence and female genital mutilation to mention the worst. The 1995 Constitution of the Land, on the other hand, unequivocally adheres the definition given by the United Nations Declaration on the Elimination of Violence against Women (DEVAW) in that under Article 35(4) states that “laws, customs and practices that oppress or cause bodily or mental harm to women are prohibited” [13].

In the revised Criminal Code of the Federal Democratic Republic of Ethiopia 2004.” Under Proclamation No.414/2004 stated that Article 564. - Violence against a Marriage Partner or a Person Cohabiting in an Irregular Union. The relevant provision of this Code (Arts. 555–560) shall apply to a person who, by doing violence to a marriage partner or a person cohabiting in an irregular union, causes grave or common injury to his/her physical or mental health [14].

In this regards, FIDO (Fayyaa Integrated Development Organization) received funding from UNFPA to implement activities on addressing gender based violence in Jimma zone of Oromiya region. The project comprises two districts of the Jimma zone namely Kersa and Sokoru district. Within these two districts 10 Kebeles [lowest administrative units] were selected to benefit from the program. Therefore, for further program implementation this baseline survey on gender based violence was conducted on Kersa and Sokoru district of the FIDA program units.

Despite increasing recognition that domestic violence is a global public health concern, population-based studies that examine violence against women and its determinants and consequences in developing countries remain scarce [15]. Therefore this population based study's main objective was to estimate the prevalence of different forms of violence against women, with particular emphasis on physical, sexual violence by intimate partners and identify factors associated with this violence.

## Methods

### Study settings and design

The study area was Jimma Zone of Oromiya region Kersa and Sokoru districts southwest of Addis Ababa. Jimma Zone, located in the southwestern part of the country, is one of the 12 Zones of Oromia Regional State. A total of 2.6 million people reside in the zone of which 89% are rural dwellers. Afaan Oromo is the official language in the Zone. Besides, Amharic, Yem and Dawro are widely spoken and the two districts were located about 260 km Southwest of Addis Ababa and 55 km North East of Jimma [16]. The districts are administratively organized into small units called Peasant Associations in rural areas and Urban Dwellers Associations in urban areas; both units are commonly referred as kebele [17]. The total populations of Sokoru district stood at 20,923 of which females accounted for 11,078 of the population. The total populations of Kersa district were 22,923 of which 11,984 of them were females. Kersa and Sokoru district, Jimma zone, Oromia regional state, southwest of Addis Ababa, capital city of the federal government of Ethiopia. Capital towns of the two districts have all weather roads that connect them with Addis Ababa and the zone capital town, Jimma. The rest of the kebeles/villages are only accessible with dry weather roods. A community based cross sectional study design was used for this baseline assessment employing interviewer-led, questionnaires based data collection techniques. This study was conducted in May 2009.

### Sample size determination

The sample size was calculated using single proportions sample size formula using Epi Info (CDC, Atlanta, U.S.A., 2005) 6.04 statistical package. The following parameters were used to calculate the sample size: proportion: since previous comprehensive survey on violence in the project sites is not available 50% prevalence on the combined GBV was considered for the calculation of sample size. Precision of 5%, and confidence limit of 95% were assumed in the sample size calculation and 10% was added to compensate for possible non-response which gave a total of 422 women considering a design effect of 2 this provided a total of 844 ever-married women.

### Sampling procedure

From the 10 Kebeles (lowest administrative unit), 2 urban and 2 rural Kebeles were selected randomly after stratification. The Kebeles or the peasant associations were similar to each other in several aspects, so selecting one from each kebele sufficed for representation. Systematic sampling technique with an interval (*K = total population of the selected kebele/desired sample size determined below*) was used to selected the household. Thus, every three (3) households were interviewed for Sokoru district while every five (5) households were interviewed for Kersa district. In case there was more than one eligible respondent (ever-married women) in a sampled household one was selected using a lottery method.

### Data collection

A validated structured questionnaire, which is adopted, from WHO [9] core questionnaire on domestic violence, prepared in Amharic language was translated into Oromifa and used to interview ever-married woman and this encompassed: socio-demographic profiles of the women, various acts of gender based violence, reasons of violence, outcomes of violence, women's view of gender based violence and ways of community interventions regarding a violence act.

### Data collection process

Data was collected through door to door face interviews with women of reproductive age (15–49 years). Written consent was obtained from respondents before the interview. Data collectors were 12th grade complete individuals who knew the local language and who had experience in data collection on similar survey. One person who knew the locality very well was assigned for each data collector for guidance and facilitation of the data collection process. Interviews were conducted individually at a convenient location for the respondents, usually outside their home but in the same compound by trained interviewers.

### Measurement

In this study, the definition of violence given by the WHO was adopted [6]. Sexual and physical violence were measured for lifetime and for 12 months prior to the survey. Physical violence was measured using 6 acts (slapped or had something thrown at her that could hurt her, pushed or shoved, hit with fist or something else that could hurt, kicked, dragged, or beaten up, choked or burnt on purpose, and perpetrator threatened to use or actually used a gun, knife, or other weapon against her) based on frequency of once or twice, a few times, or many times [6]. Sexual violence was measured using three acts (physically forced to have sexual intercourse when she did not want to, had sexual intercourse when she did not want to because she was afraid of what partner might do and was forced to do something sexual that she found degrading or humiliating). The lifetime prevalence of partner violence was then defined as the proportion of ever-married women who reported having experienced one or more acts of physical or sexual violence by a current or former partner at any point in their lives. Current prevalence is the proportion of ever-married women reporting at least one act of physical or sexual violence during the 12 months before the interview. Controlling behaviors by an intimate partner was measured using four questions keeps her from seeing friends; restricts her contact with family; insists on knowing where she is at all times; gets angry if she speaks with other men. If one of the above is mentioned to occur the partner is designated controlling.

### Statistical analysis

Data was checked in the field by supervisors and principal investigator and entered using Epi DATA version 3.1 and exported into STATA version 10.1 software and SPSS version 12.0.1 for windows for data analysis. Data exploration was done to see if there were odd observations and then summary statistics and graphical techniques were used to describe the data. Proportions and cross-tabulations of selected characteristics were used to describe the study participants and the outcome characteristics. Logistic regression analysis was done to identify factors associated with violence. The dependent variable “Experience of violence in the past 12 months” was used for bivariate and multivariate analysis. To identify factors associated with physical and sexual violence bivariate analysis was done, variables found to be associated (P≤0.05) with the outcome variable were entered together into logistic regression model. The variables included were age of woman, age of partner, educational status of women and partners, residence (urban versus rural), duration of relationship, household income, religion, occupational status of women, age at first marriage, partner control behavior (controlling versus not controlling), a woman can do nothing if husband wants a girlfriend (yes, no). Finally two logistics models were produced for physical violence and sexual violence.

### Ethical considerations

This study followed ethical recommendations for research on domestic violence by WHO and also other ethical requirements related to research on human beings [18]. Respondents had been provided with an informed consent and participation to each part or whole part of the interview was on voluntary basis. The names and any personal identifiers were not used in the survey questionnaires. Each participant's verbal consent was obtained regarding their participation in the study after assuring confidentiality. Due to the large number of individuals that were surveyed and high illiteracy, it was considered impractical to obtain written consent from each study participant. The core protocol and research instruments were approved by the Oromia Regional Health Bureau Ethical Review Committee. Participants involved in the study were fully informed about the nature of the study, research objectives, and confidentiality of the data. Interviewers orally provided contact information for organizations that address violence and conflict within marriage to all women. If any respondent ever experienced violence and sought help (either required counseling or other services) then interviewers facilitated access to an appropriate service facility or referred her to the nearest health centre.

## Results

### Socio-demographic profiles of the survey participants

A total of 845 women of reproductive age group were successfully interviewed with a response rate of 100%. The observed rate of response in this baseline study could be partially due to the smooth relationship established between the study team and the community in the study area which was started by brief and effective communication made with the leaders at the district level which was extended down to the leaders of peasant associations selected for the study.

Majority of the women were Muslim 725(85.8%) by religion, Oromo by Ethnicity 733(86.1%) and housewives by occupation 570(69.4%). The mean age (standard deviation) of the women were 30.6(7.76) while for their spouses this was 38.4(10.9). The majority of the women had had no formal education 538(66.5%). The mean (standard deviation) age at first marriage was found to be 17.8(3.6). The average monthly income of the women was 14.35 USD (Exchange rate 1 USD = 13 Ethiopian Birr (ETB)) (Table 1).

**Table 1:** Socio-demographic characteristics of survey participants, Southwest Ethiopia May 2009.

Characteristics	Number (%)
Residence	
Urban	484(57.3)
Rural	360(42.7)
Age of the women Mean(SD)	30.6(7.76)
Age of the partner Mean(SD)	38.4(10.9)
Education of the women	
Illiterate	561(69.1)
Read and write	95(11.7)
Primary	114(14.0)
Secondary and above	42(5.2)
Education of the partner	
No education	328(51.4)
Read/write/no formal education	137(21.5)
Formal education	173(27.1)
Religion	
Muslim	725(85.8)
Orthodox Christian	84(9.9)
Protestant	30(3.6)
Catholic	6(0.7)
Ethnicity	
Oromo	733(86.1)
Amhara	34 (4.0)
Yem	74 (8.7)
Others*	10(1.2)
Occupation of the women	
House wife	570(69.4)
Farmer	124 (15.1)
Trader	54 (6.6)
Laborer	35(4.3)
Others♀	38(4.6)
Mean Monthly income	14.35
Age at first marriage Mean(SD) and Median	17.8(3.6), 17.0

* *Somale, Guraghe,*

♀ *student, government employee.*

(Exchange rate 1 USD = 13 Ethiopian Birr (ETB)).

### Prevalence of intimate partner violence

The proportion of women ever reporting either sexual or physical partner violence, or both, 64.7%(95%CI: 61.4%–67.9%). The proportion women ever reporting physical violence is 41.1%(95%:.8–44.5), while the proportion of women reporting sexual violence ever is 50.1%(95%CI: 46.7%–53.4%). The physical violence by the partner was considerably less prevalent than sexual partner violence.

Regarding the proportion of ever reporting intimate partner violence within the previous 12 months, 41.5%(95%CI: 38.2%–44.8%) reported physical or sexual violence, or both, in the past year. Whereas 28.8%(95%CI: 25.8%–31.9%) and 28.0%(95%CI: 25.1–31.1) reported physical and sexual violence in the past year respectively (Table 2). 

**Table 2:** Prevalence of physical, sexual or both violence by intimate partner in Southwest Ethiopia.

Acts of Violence	Ever	95%CI	Current	95%CI
Physical Violence	340(41.1)	37.8–44.5	245(28.8)	25.8–31.9
Sexual Violence	415(50.1)	46.7–53.4	238(28.0)	25.1–31.1
Physical or sexual or both	536(64.7)	61.4–67.9	353(41.5)	38.2–44.8

There was a substantial overlap between physical and sexual violence by intimate partners, in our study 219(26.4%) women who had ever experienced any violence reported both physical and sexual violence. There were more women (23.6%) who reported only sexual violence than only physical violence (14.6%).

### Perceived outcome of violence

Among women who reported sexual violence during last year, they were asked about perceived consequences of the sexual violence reported. Most (38.6%) reported unwanted pregnancy (Table 3).

**Table 3:** Reported outcomes of current sexual violence among ever-married woman, Southwest Ethiopia.

Outcome (N = 215)	Number	Percent
Unwanted pregnancy	83	38.6
Physical injury	23	9.7
Abandonment	13	5.5
Genital Ulcer	8	3.4
Genital Discharge	3	1.3
Genital itching	2	0.8
Discontinued education	12	5.0
HIV/AIDS	6	2.5
Others	65	30.2

### Perceived reasons for conflict

As depicted in Table 4 most (20.4%) mentioned that there was no specific reason for the quarrel. Considerable women indicated a rage of reasons for the conflict. Economic reasons were the most frequent followed by power relation and partner being drank (Table 4).

**Table 4:** Reasons for quarrel which leads to violence among ever-married women who ever reported physical, sexual or both violence, in Southwest Ethiopia.

Reasons(n = 525)	Frequency	Present
No specific reason	107	20.4
When he has shortage of money	82	15.6
When I didn't obey his orders	62	11.8
When he is drank	50	9.5
When he feels jealous	41	7.8
When there is shortage of food	23	4.4
When I inquire about his infidelity	23	4.4
When there is problem in the family	18	3.4
When he has problem on job	12	2.3
When I refuse sex	8	1.5
Others	99	18.5

### Factor associated with violence in the past 12 month

Two factors were associated with physical violence after adjusting for variables indicated in Table 5. Those women who did not believe a wife could do anything if a husband wants a girlfriend were more likely to report physical violence (OR = 3.4; 95%CI: 1.5–7.6). Woman with a controlling partner are more likely to report physical violence (OR = 6.4; 95%CI: 3.8–10.8).

**Table 5:** Factors associated with current physical violence among ever-married women Southwest Ethiopia.

Variable	Current physical violence		Unadjusted OR (95%CI)	Adjusted OR (95%CI)
Education woman	Yes	No		
Illiterate	140(25.0)	421(75.0)	0.33(0.17–0.62)	0.63(0.25–1.58)
Read and write	32(33.7)	63(66.3)	0.51(0.24–1.06)	1.23(0.43–3.54)
Primary	43(37.7)	71(62.3)	0.61(0.29–1.24)	0.72(0.29–1.80)
Secondary and above	21(50.0)	21(50.0)	1.0	1.0
Husband education				
Illiterate	80(24.4)	248(75.6)	0.29(0.17–0.50)	0.61(0.28–1.31)
Read and write	30(21.9)	107(78.1)	0.26(0.14–0.48)	0.38(0.17–0.84)
Primary	39(38.6)	62(61.4)	0.58(0.31–1.07)	0.69(0.33–1.46)
Secondary and above	37(52.1)	34(47.9)	1.0	
Age of husband				
<30	56(25.6)	163(74.4)	0.43(0.23–0.79)	1.03(0.54–1.94)
30–45	130(32.5)	270967.5)	1.15(0.76–1.75)	0.81(0.47–1.41)
>45	45(30.8)	101(69.2)	1.0	1.0
If husband get girlfriend woman can not do anything				
Yes	233(31.6)	505(68.4)	3.8(2.1–7.2)	**3.4(1.5–7.6)**
No	12(10.6)	101(89.4)	1.0	1.0
Occupation of wife				
Housewives	150(26.3)	420(73.7)	0.66(0.48–0.91)	0.94(0.58–1.52)
Others	88(35.1)	163(64.9)	1	1.0
Controlling				
Yes	37(11.2)	292(88.8)	5.56(3.78–8.17)	**6.4(3.8–10.8)**
No	207(41.3)	294(58.7)	1.0	1.0
Residence				
Urban	171(35.3)	313(64.7)	2.15(1.6–2.9)	1.27(0.75–2.14)
Rural	73(20.3)	287979.7)	1	1

Similarly two factors were associated with sexual violence after adjusted for variables reported in Table 6. Housewives are less likely to report sexual violence than working women (OR = 0.57; 95%CI: 0.36–0.89). Concurrences with physical violence women with controlling partner were more likely to report sexual violence than their counterpart (OR = 4.7; 95%CI: 2.8–7.9).

**Table 6:** Factors associated with current sexual violence among ever-married women Southwest Ethiopia.

Variable	Current physical violence		Unadjusted OR (95%CI)	Adjusted OR (95%CI)
Education woman				
Illiterate	139(24.8)	422(75.2)	0.33(0.17–0.62)	0.74(0.30–1.80)
Read and write	32(33.7)	63(66.3)	0.51(0.24–1.06)	1.35(0.49–3.76)
Primary	37(32.5)	77(67.5)	0.61(0.29–1.24)	1.03(0.42–2.54)
Secondary and above	16(38.1)	26(61.9)	1.0	1.0
Husband education				
Illiterate	64(19.5)	264(80.5)	0.47(0.27–0.83)	1.04(0.49–2.19)
Read and write	37(27.0)	100(73.0)	0.72(0.39–1.35)	1.11(0.52–2.4)
Primary	37(36.6)	64(63.4)	1.13(0.59–1.14)	1.05(0.51–2.18)
Secondary and above	24(33.8)	47(66.2)	1.0	
If husband get girlfriend woman cannot do anything				
Yes	213(28.9)	525(71.1)	3.8(2.1–7.2)	1.33(0.69–2.59)
No	25(22.1)	88(77.9)	1.0	1.0
Occupation of wife				
Housewives	125(21.9)	445(78.1)	0.39(0.28–0.54)	**0.57(0.36–0.89)**
Others	105(41.8)	146958.2)	1	1.0
Controlling				
Yes	39(11.9)	290(88.1)	4.82(3.29–7.04)	**4.7(2.8–7.9)**
No	197(39.3)	304960.7)	1.0	1.0
Residence				
Urban	174(36.0)	310(64.0)	2.69(1.9–3.7)	1.56(0.94–2.59)
Rural	62(17.2)	298(82.8)	1	1

## Discussion

In this study 64.7% of ever-married women reported physical or sexual violence, or both, by an intimate partner at some point in their lives. Sizable (41.5%) women reported physical or sexual violence, or both, by a partner within the 12 months before the study. Partner controlling behavior was found to be associated with physical violence and Housewives and women without controlling partner were less likely to report sexual violence.

The life time prevalence of any type of violence in this study was higher than northwest Ethiopia (50.8%) [10] and southwest Ethiopia (51.8%) [11]. It was found to be less than central Ethiopia (70.9%) as reported by the WHO multi-county study [9]. In the previous 12 months, a prevalence of 41.5% was obtained which is less than that of central Ethiopia (53.7%) [9]. This could be attributed by difference in the year of the survey. The central Ethiopian study was conducted in 2002. In recent years, there are a number of community mobilization activities implemented regarding ending gender based violence in the country which might have a positive impact on reducing violence.

In majority of the cases there was an overlap between physical and sexual violence. In our study more sexual violence was reported than physical violence, and this is consistent with previous study from Ethiopia, Bangladesh, and Thailand [9] other studies that have reported similar finding on sexual violence and physical violence among women [19], [20].

More interestingly, in South African studies, results demonstrate the association between physical partner violence against women and HIV sero-status. In the past year, 30.7% (362/1180) of men had been physically violent towards an intimate partner more than once. The model of factors associated with having HIV showed men under 25 years who had been physically violent towards partners were more likely to have HIV than men under 25 who had not [7]. From a total of 168 women who were assisted in the project, emotional (139, 82.7%), physical (115, 68.5%), sexual (72, 42.9%) and financial abuse (72, 42.9%) were common and 114 (67.9%) had a high to severe risk of harm [8].

According to the Ethiopian Demographic and health Survey 2005 [21] , 85% of women believe husbands are justified in beating their wife for at least on reason. Similarly, in rural southwest Ethiopia, women who experienced IPV were more likely than the non-abused women to believe that a man could be justified to hit his partner when she failed to complete her work or when she did not obey him [11]. This indicates the level of acceptance for physical violence in Ethiopia which might result underreporting of it, while sexual punishments are unacceptable.

The prevalence (41.1%) of physical violence in lifetime is lower than previous studies [9], [12] but 81% of the women reported severe physical violence. This can highlight the possibility of underreporting of physical violence in Ethiopia due to the context in which it is considered as an acceptable means for husbands to control or chastise their wives [21].

Those women who believed a wive could do anything if their husbands wanted girlfriends were more likely to report physical violence. This could be due to internalizing the social norms and acceptance of gender based violence as normal. Housewives are at lower risk of sexual violence than working women. This could be due to the exposure of women to the larger society men might be violent on working women so as to prevent women from working outside the home as a way of isolating and controlling them. In addition, involvement of women in economic activity might be considered as a challenge in power sharing with man hence acted more provocatively than protective. Similar findings were reported in other study [22].

Partner controlling behavior is associated with reported violence. Women with controlling partner are more likely to report both physical and sexual violence. This is consistent with previous studies [9], [23], [24]. However there are arguments about controlling behavior, whether it is contributing factor for violence or part of the violence acts. Whatever the case our result reinforces that the importance of partners controlling behavior in violence.

The cross-sectional nature of the study limits on the casual pathway or temporal relationships cannot be inferred. Recall bias could also be another limitation, to minimize these we have used one year recall period in addition to the lifetime recall. Finally violence related issues are very sensitive and there might be non-disclosure which might lead to under-reporting. The strength of the study includes using standardized instrument and rigorous training of data collectors, high response rate and inclusion of urban and rural communities.

### Conclusion

This study indicated that marriage should not be considered as a safe haven. IPV remains significant public health problem among ever-married women in Southwest Ethiopia. Majority (64.7%) of ever-married women reported physical or sexual violence, or both, by an intimate partner at some point in their lives. Our analysis indicated that partner controlling behavior increases the odds of violence among ever-married women. Interventions targeting controlling men might help in reducing IPV.

Therefore context specific interventions supported by formative assessment such as further prospective longitudinal studies among ever-married woman are important to identify predictors and study the dynamics of violence over time and addressing IPV in comprehensive manner including the medical and psychiatric consultation (depression, anxiety, PTSD), bounding with legal protection and advise within an intervention effort is recommended as to effectively reduce IPV among ever-married women. Here again, the study shows a clear description and highlight for policy makers and FIDO project to intervene on operational area of the project.
